# The role of herpes simplex virus infection in the etiology of head and neck cancer–a Mendelian randomization study

**DOI:** 10.3389/fimmu.2024.1278327

**Published:** 2024-08-05

**Authors:** Ming Yan, Li-yuan Xiao, Martin Gosau, Ralf Smeets, Hong-chao Feng, Simon Burg, Ling-ling Fu, Reinhard E. Friedrich

**Affiliations:** ^1^ Department of Oral and Maxillofacial Surgery, Guiyang Hospital of Stomatology, Guiyang, China; ^2^ Department of Oral and Maxillofacial Surgery, University Medical Center Hamburg-Eppendorf, Hamburg, Germany; ^3^ Department of Oral and Maxillofacial Surgery, Division of Regenerative Orofacial Medicine, University Medical Center Hamburg-Eppendorf, Hamburg, Germany

**Keywords:** head and neck cancer, herpes simplex virus, Mendelian randomization, causal effect, hsv, oral and oropharyngeal cancer

## Abstract

**Introduction:**

Head and neck cancer (HNC) is a complex disease, and multiple risk factors can lead to its progression. Observational studies indicated that herpes simplex virus (HSV) may be correlated with the risk of HNC. However, the causal effects and direction between them were still unclear.

**Methods:**

This study utilized a Mendelian randomization (MR) approach for causality assessment between HSV infection and Head and neck cancer based on the latest public health data and Genome-Wide Association Study (GWAS) data. The causal effects were estimated using IVW, weighted median, and MR-Egger. A reverse MR analysis was subsequently performed. Cochrans *Q* test, MR‐Egger intercept test, leave one out analysis, and the funnel plot were all used in sensitivity analyses.

**Results:**

Genetically predicted higher level of HSV-1 IgG was causally related to HNC (OR=1.0019, 95%CI=1.0003–1.0036, p=0.0186, IVW) and oral and oropharyngeal cancer (OR=1.0018, 95%CI=1.0004–1.0033, p=0.0105, IVW). The reverse MR analysis did not demonstrate a reverse causal relationship between HSV and HNC. However, HSV-2 infection was not causally related to HNC data and oropharyngeal cancer data. Sensitivity analysis was performed and revealed no heterogeneity and horizontal pleiotropy.

**Conclusion:**

Collectively, a significant association was noted between HSV infection and increased risk of HNC, providing valuable insights into the etiology of this malignancy. Further in-depth study is needed to validate these findings and elucidate the underpinning mechanisms.

## 1 Introduction

Head and neck cancer (HNC) is a complicated and multi-factorial disease that consists of a heterogeneous group of malignant tumors in the upper respiratory tract, covering the oral cavity, pharynx, throat, and nasal cavity (1). It is an important global health burden and is responsible for a considerable proportion of morbidity and mortality relevant to cancers on global scale. Despite advances in treatment modalities, the prognosis of this malignancy is still poor, which emphasizes the demand for a deeper understanding of its etiology and identification of new risk factors (2).

Herpes simplex virus (HSV) infection is triggered by two distinct serotypes, HSV-1 and HSV-2, showing a high prevalence in the general population. HSV-1 mainly infects the lip and mouth areas, resulting in recurrent oral lesions, commonly referred to as cold sores, while HSV-2 primarily causes genital herpes (3). In addition to the well-known manifestations, HSV infection is also linked to multiple diseases, including cancer. Several studies have discussed the potential link between HSV infection and the progression of HNC and have proposed direct and indirect mechanisms (4).

Previous epidemiological investigations have reported the relationships between HSV infection and HNC, especially oropharyngeal cancer. However, the nature of these relationships and potential causal associations remain undefined (5). Observational studies have inherent limitations, such as confounding factors and reverse causality, which hinders their ability to definitively establish causality. Rigorous and innovative research designs are required to overcome these challenges and clarify the causal role of HSV infection in HNC (6).

Mendelian randomization (MR) analysis, an instrumental variable approach with genetic variants serving as instrumental variables (IVs), represents a powerful tool for assessing causality in epidemiological studies (7). Though random assignment of genetic variants during the gamete formation process and their correlations with relevant exposures, the MR analysis can provide strong evidence for causality (8). In terms of HSV infection and HNC, the MR analysis offers a unique opportunity to overcome the limitations of observational studies and clarify the potential causal mechanism of their associations (9).

Therefore, in this study, a comprehensive MR analysis was carried out to explore the causality between HSV infection and the development of HNC, particularly oropharyngeal cancer. By utilizing large-scale genomic data and HSV infection-related genetic tools, we probed into whether HSV-1 and HSV-2 infections were causally relevant to the risk of HNC (10).

The results of this study were of great significance for understanding the etiology of HNC and may pave the way for targeted interventions to attenuate the burden of this malignancy. By elucidating the causal implication of HSV infection in HNC, we could identify prevention strategies and treatments specifically targeting HSV-related pathways (11). Ultimately, these insights may contribute to the improvement of patient prognosis, early detection, and personalized management of HNC (12).

## 2 Materials and methods

To study the causal relation between HSV and HNC, we conducted a bidirectional two-sample Mendelian randomization (TSMR) study in accordance with the latest STROBE-MR (Strengthening the Reporting of Observational Studies in Epidemiology Using Mendelian Randomization) guidelines (13). MR is a powerful analytical method that assesses the causality in observational studies using genetic variants as IVs.

The TSMR analysis consisted of two major procedures: estimating the genetic association with exposure (HSV infection) and estimating the genetic association with outcome (HNC). These estimated values were then combined for assessing the causal impact of the exposure on the outcome (14).

Three key assumptions must be met to ensure the validity of MR analysis:

Strong IV association: the selected IVs should be closely linked to the exposure variable (HSV infection). We identified genetic variants that had previously been validated and demonstrated to be strongly associated with HSV infection on the ground of large-scale genome-wide association studies (GWAS) or other credible sources (15).
Independence of IVs: the IVs adopted in the analysis should be independent of any confounding factors that might affect the outcome (HNC). We carefully selected IVs that had been proven to be independent of known confounding factors through extensive literature review and consultation with experts in the field (16).
Exclusion restriction assumption: IVs should affect the outcomes only *via* the association with the exposure variable (HSV infection). This assumption guaranteed that IVs would not have a direct impact on outcomes independent of their impact on HSV infection (17).

To evaluate the strength of IVs and prevent the impact of weak instruments on causality, we calculated statistical values using the formula: F=β^2^_exposure/SE^2^_exposure. Weak IVs were defined as F<10, indicating limited statistical power to reliably estimate the causal effect (18).

The data adopted in this article were publicly available to researchers worldwide, so no additional ethical approval and informed consent were required. We gained the summary statistics of necessary genetic associations between HSV infection and HNC from publicly available GWAS datasets and consortia (19).

**Figure 1:**
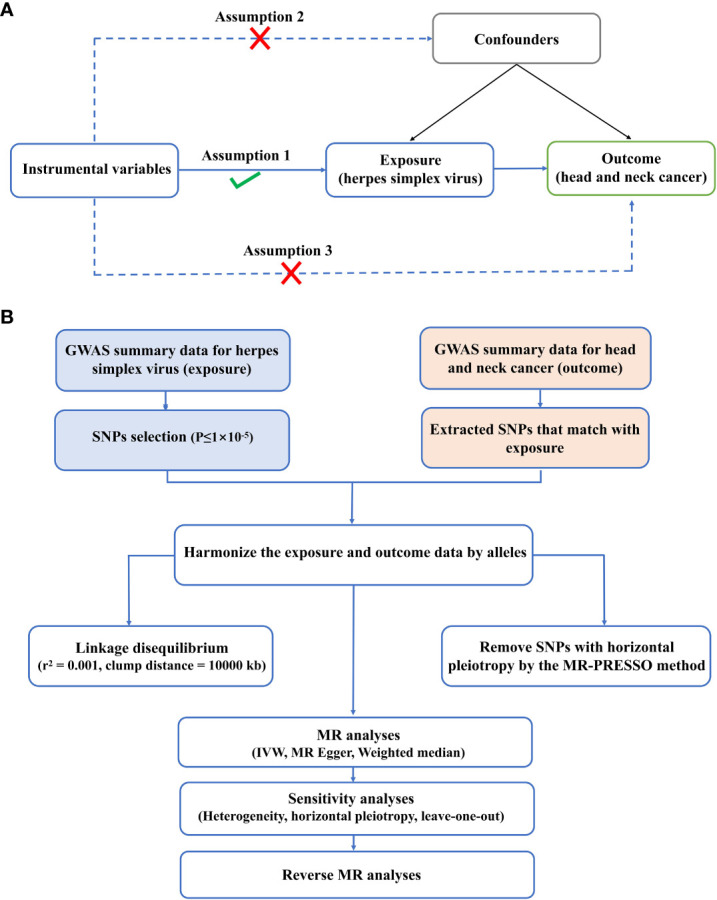
Experimental design and assumptions. **(A)** Three key assumptions of MR analysis. **(B)** Flow chart of experimental design.

According to the latest STROBE-MR guidelines, this paper conducted a bidirectional TSMR study to observe the causal relation between HSV and HNC. MR study must meet three principal assumptions: IVs should be strongly linked to exposure; (2) IVs should be independent of any possible confounders; (3) IVs affected the outcomes only *via* the exposure (**Figure 1A**). To avoid the impact of weak IVs on causality, the statistical values of IVs were calculated based on the formula F=β ^2^ exposure/SE ^2^ exposure. A weak IV was defined if F<10. The data utilized in the present study were publicly available to global researchers (20). Hence, no additional ethical approval and informed consent were required. The procedures of the experimental design are presented in **Figure 1B**.

### 2.1 HSV infection data

Butler et al. conducted a GWAS analysis on infectious pathogens in 2020, which enrolled 8735 individuals, and serum samples were provided for the detection of antibody levels against a variety of antigens, including HSV IgG1 antibody and IgG2 antibody. Antibody detection was done using a Luminex 100 platform (Luminex Corporation, Austin, TX, USA) at a dilution of 1:1000 using a fluorescent bead-based multiplex serology technique. This approach provided the median fluorescence intensity (MFI), which allowed standardized quantification of antibodies in the samples obtained by detecting the fluorescence signal that was emitted by the analyte-trap complex. This approach and the selection of seropositive threshold had been validated for multiple infectious pathogens. The MFI seropositive threshold of the HSV IgG1 antibody and IgG2 antibody was 150. There were 6199 cases diagnosed as HSV-1 positive and 1382 cases diagnosed as HSV2 positive. The study of Butler et al. was currently the largest GWAS study on HSV serological tests (21).

The database used to obtain HSV information was from the study conducted by Butler et al. in 2020. In their study, Butler et al. carried out a GWAS analysis of infectious pathogens, including HSV, using a dataset consisting of 8735 individuals who offered serum samples for antibody detection against various antigens (22).

The fluorescent bead-based multiplex serology technology was adopted for antibody detection on the Luminex 100 platform manufactured by Luminex company (Austin, TX, USA). Serum samples were diluted at a ratio of 1:1000, and antibody levels were measured using MFI. MFI provided standardized quantification of the antibody concentration in the samples *via* the detection of the fluorescence emitted by the analyte-trap complex (23).

To determine the seropositivity of HSV, a specific threshold was established according to the effective criteria of multiple infectious diseases. In the present study, the seropositive threshold of the HSV IgG1 antibody and IgG2 antibody was set at 150 MFI. Therefore, 6199 individuals were diagnosed as HSV-1 positive, and 1382 were diagnosed as HSV-2 positive. It was worth noting that this GWAS analysis on HSV serological detection conducted by Butler et al. represented the largest such study to date.

Using the comprehensive dataset provided by Butler et al., this study adopted the information on HSV serology positivity to explore the causal relation between HSV infection and the development of HNC. The large sample size and validated serological detection methods used in the study of Butler et al. contributed to the robustness and reliability of our analysis and enhanced the validity of the research results presented in this paper.

### 2.2 HNC data

UK Biobank is currently the largest GWAS database in the world, and the research population involves volunteers across the UK. The datasets, including HNC, Laryngeal cancer, Oral and oropharyngeal cancer, Oral cavity cancer, and Oropharyngeal cancer, were downloaded from UKB at https://biobank.ndph.ox.ac.uk/ukb/search.cgi (24), Details and data sources are given in **Table 1**.

**Table 1:** General description of data sources involved in the MR analysis.

Year	Trait	Consortium	Sample size	Case	Number of SNPs	Population
2021	Head and neck cancer	UK Biobank	373122	1,106	9655080	European
2021	Oral and oropharyngeal cancer	UK Biobank	372855	839	9185233	European
2020	HSV-1 IgG	Butler-Laporte G	9724	8735	9170312	European
2020	HSV-2 IgG	Butler-Laporte G	9724	8535	9170312	European

MR, Mendelian randomization.

### 2.3 Selection of IVs

Linkage disequilibrium (LD) thresholds were adopted for the extracted SNP (r^2^ < 0.001, 10000 kb) to avoid the effect of LD so as to ensure independence between IVs at each exposure. Palindromic alleles were eliminated. Additionally, the F statistic was utilized to assess the strength of the IV-exposure correlation. A value of F statistic > 10 was deemed to be strong enough to avoid weak IV-induced bias. In order to satisfy the second assumption of MR, we further searched these SNPs in the PhenoScanner database (http://www.phenoscanner.medschl.cam.ac.uk/) and excluded SNPs related to other putative confounding factors (smoking, drinking frequency, etc.).

### 2.4 TSMR analysis

TSMR analysis was employed to analyze the causality between HSV infection on head and neck squamous carcinoma. MR methods included inverse variance weighted (IVW), MR-Egger, and weighted median (WM). As the most common MR method that could estimate the causal effect by integrating the ratio estimate of each SNP, the IVW method was the major analysis method used in this study. The MR-Egger intercept test could evaluate the horizontal pleiotropy in the MR analysis through the intercept of MR-Egger regression (horizontal pleiotropy was defined as p<0.05). After sequentially eliminating the SNP locus, the leave-one-out analysis used the remaining SNP loci for MR analysis to test whether there was bias caused by a specific SNP locus, and it adopted the IVW method for calculation. In MR analysis, the symmetry of the funnel plot was able to evaluate the reliability of associations. We applied the IVW method to assess the influence of all genetic variables on the outcomes. The Cochran Q test of IVW was employed to evaluate the heterogeneity between SNPs, and p>0.1 suggested no heterogeneity among genetic tools. Mendelian randomization-pleiotropy residual sum and outlier (MR-PRESSO) consisted of three parts: i) detection of horizontal pleiotropy; ii) correction of pleiotropy by eliminating detection outliers (genetic variants with horizontal pleiotropy); iii) comparison of the differences in causal relations before and after correction. Eventually, reverse TSMR analysis was performed with HNC as exposure and HSV infection as an outcome. MR analyses were accomplished by the “TwoSampleMR” and “MR-PRESSO” packages (R version 4.1.2). Power analysis was performed using mRnd (https://shiny.cnsgenomics.com/mRnd/). All analyses were based upon public data with no need for additional ethical approval and informed consent of participants since these had been obtained at the initial release (25).

## 3 Results

### 3.1 HSV-1 infections might related to HNC

**Figure 2:**
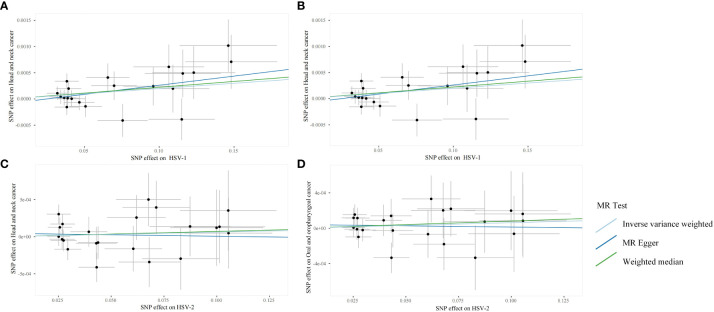
Scanner plots for the two-sample Mendelian randomization analyses. **(A)** HSV-1 and head and neck cancer; **(B)** HSV-1 and Oral and oropharyngeal cancer; **(C)** HSV-2 and head and neck cancer; **(D)** HSV-2 and Oral and oropharyngeal cancer.

Through the aforementioned screening conditions, 44 SNPs were found to be significantly associated with HSV infection, including 22 in HSV-1 and 22 in HSV-2, with F statistic values >10. No confounding factors of HNC were found after searching at Phenoscannerv2. The details for SNPs are described in **Supplementary Table 1**. As revealed by the positive MR analysis, HSV-1 IgG was causally related to HNC (OR=1.0019, 95%CI=1.0003–1.0036, *p*=0.0186, IVW), and oral and oropharyngeal cancer (OR=1.0018, 95%CI=1.0004–1.0033, *p*=0.0105, IVW) (**Table 2**). All causal effects of HSV on HNC assessed by the three MR methods were visualized in the scatter plot, wherein a slope greater than zero indicated a positive correlation (**Figure 2**). However, HSV-2 infection was not causally related to HNC data and oropharyngeal cancer data. The results of *post-hoc* power calculations were shown in **Supplementary Table 2**.

**Table 2:** TSMR analysis of the causal relation between HSV and head and neck cancer.

Exposures	Outcomes	SNPs	Methods	OR	95% CI	*p*
HSV-1 (IgG)	Head and neck cancer	21	MR-Egger	1.0034	0.9996–1.0073	0.0909
			Weighted median	1.0022	0.9998–1.0045	0.0651
			IVW	1.0019	1.0003–1.0036	0.0186
	Oral and oropharyngeal cancer	21	MR-Egger	1.0033	1.0008–1.0066	0.0584
			Weighted median	1.0016	0.9995–1.0037	0.1248
			IVW	1.0018	1.0004–1.0033	0.0105
HSV-2 (IgG)	Head and neck cancer	22	MR-Egger	0.9996	0.9949–1.0042	0.8724
			Weighted median	1.0007	0.9977–1.0037	0.6417
			IVW	1.0006	0.9985–1.0027	0.5621
	Oral and oropharyngeal cancer	22	MR-Egger	0.9997	0.9956–1.0037	0.8951
			Weighted median	1.0008	0.9984–1.0031	0.4997
			IVW	1.0006	0.9988–1.0024	0.5025

HSV, herpes simplex virus; IVW, inverse-variance weighted; OR, Odds ratio; CI, confidence interval.

### 3.2 Sensitivity analysis revealed no heterogeneity and horizontal pleiotropy

**Figure 3:**
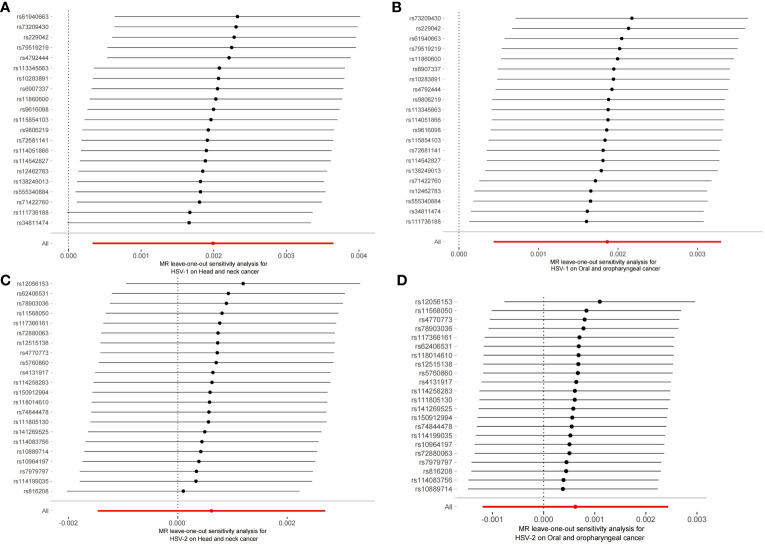
Plots of leave-one-out analyses for the two-sample Mendelian randomization analyses. **(A)** HSV-1 and head and neck cancer; **(B)** HSV-1 and Oral and oropharyngeal cancer; **(C)** HSV-2 and head and neck cancer; **(D)** HSV-2 and Oral and oropharyngeal cancer.

**Figure 4:**
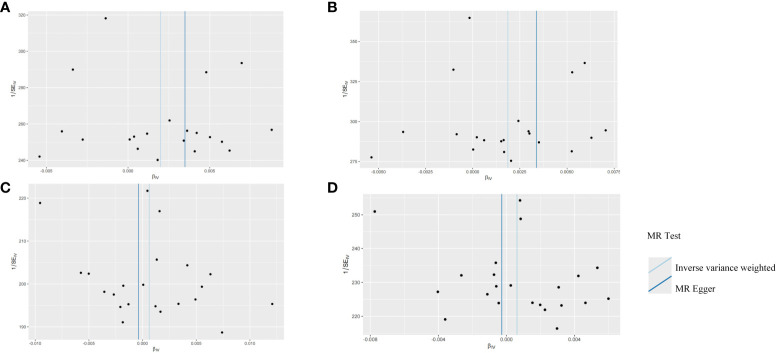
Funnel plots for the two-sample Mendelian randomization analyses. **(A)** HSV-1 and head and neck cancer; **(B)** HSV-1 and Oral and oropharyngeal cancer; **(C)** HSV-2 and head and neck cancer; **(D)** HSV-2 and Oral and oropharyngeal cancer.

The robustness of the aforementioned causal associations was validated based on the data from the sensitivity analysis. The heterogeneity test revealed no heterogeneity in the MR analysis (Cochran’s Q statistic, p>0.05). The MR-Egger regression analysis failed to provide evidence for horizontal pleiotropy (MR-Egger intercept<0.01, p>0.05). The MR-PRESSO global test suggested that no noticeable outliers were able to drive the causal effect (p>0.05) (**Table 3**). The leave-one-out analysis further displayed no single SNP driving the causal effect (**Figure 3**), and the symmetry data of the funnel plot exhibited no significant heterogeneity (**Figure 4**).

**Table 3:** Results of the sensitivity analysis.

Exposure	Outcome	Heterogeneit			MR‐Egger regression		MR-PRESSO
		Method	Q	Q-Pvalue	Intercept	p_intercept	Global test P
HSV-1	Head and neck cancer (UKB)	MR-Egger	19.69031	0.413425	8.67618E-05	0.407185	0.95
		IVW	20.43493	0.431035			
	Oral and oropharyngeal cancer (UKB)	MR-Egger	16.94983	0.593267	8.76034E-05	0.330346	0.99
		IVW	17.94784	0.590844			
HSV-2	Head and neck cancer (UKB)	MR-Egger	20.25135	0.442309	4.50223E-05	0.642206	0.09
		IVW	20.4767	0.491272			
	Oral and oropharyngeal cancer (UKB)	MR-Egger	12.59259	0.894172	4.02205E-05	0.632071	0.46
		IVW	12.82903	0.914494			

In light of the results of the reverse MR analysis, no significant causal relations were noted between HNC and oropharyngeal cancer and HSV infection.

## 4 Discussion

According to the IVW genetically predicted HSV-1 was found to be positively associated with HNC risk, especially oral and oropharyngeal cancer. The methods of Inverse Variance Weighting (IVW) are deemed dependable in instances where Mendelian randomization analyses are unaffected by pleiotropy and heterogeneity. Complementarily, the Weighted Median (WM) approach is frequently employed alongside IVW. This WM technique prioritizes the estimation of causal effects by weighing and ranking the effect estimates from all instrumental variables, ultimately determining the causal effect based on the median value. In large sample sizes, the stability of each instrumental variable’s estimate enhances the reliability of the median estimate. Conversely, in smaller samples, the median may exhibit greater variability due to the more volatile nature of the estimates. This paper, which investigates HSV-1 and HSV-2, operates with smaller sample sizes, potentially leading to more fluctuating results. Consequently, the primary focus of this study is on the outcomes derived from the IVW method.

Our MR analysis offered convincing evidence supporting the role of HSV-1 as a hazardous factor for HNC, especially oral and oropharyngeal cancer. However, no causal association was observed between HSV-2 infection and HNC, including oral and oropharyngeal cancer. These findings provided valuable insights into the etiology of these malignancies and were of great significance for clinical practice and future research (26).

The association between HSV-1 and HNC was consistent with previous epidemiological studies, which reported a higher rate of HSV-1 infection in patients with oral and oropharyngeal cancer compared with controls (27). HSV-1 is a common virus that mainly infects oral and oropharyngeal mucosa, resulting in recurrent oral ulcers or cold sores. The virus establishes latency in the trigeminal ganglion and can reactivate periodically, leading to virus shedding and potential transmission to others (28).

The mechanism by which HSV-1 leads to the progression of HNC is multifactorial and complex. HSV-1 infection will trigger a series of immune responses, resulting in the activation of various inflammatory mediators. Sustained or repeated viral replication and shedding can lead to chronic inflammation in the oral and oropharyngeal mucosa, which in turn will promote tissue damage and genetic changes, key events in the initiation and development of cancers. Inflammatory mediators, encompassing cytokines, chemokines, and growth factors, are released in the immune response to HSV-1 infection, creating an environment conducive to cell transformation. These molecules can induce DNA damage, disrupt cell signaling pathways, and accelerate abnormal cell proliferation and survival (29). Additionally, chronic inflammation induces the release of reactive oxygen species (ROS) and reactive nitrogen species (RNs), possibly resulting in DNA damage and genomic instability, which is a hallmark of cancer development. HSV-1 has evolved several strategies to evade and modulate the host immune response, which may have a significant impact on cancer development (30). Through multiple immune evasion mechanisms, such as interfering with antigen expression, this virus can down-regulate the major histocompatibility complex (MHC) molecules and inhibit the activation and function of immune cells, including T cells and natural killer (NK) cells. Dysregulation of immune checkpoints, such as PD-1 and CTLA-4, is another key mechanism by which HSV-1 may facilitate carcinogenesis (31). HSV-1 infection can up-regulate immune checkpoint molecules on T cells, which causes cell dysfunction and impaired anti-tumor immune response. Immune checkpoint ligands, such as PD-L1 expressed by infected or malignant cells, can interact with immune checkpoint receptors on T cells, which further inhibits the immune response and stimulates the immune evasion of virus or tumor cells (32). HSV-1 infection disrupts multiple pathways engaged in cell proliferation, apoptosis, and immune response. Multiple virus-encoded proteins can manipulate the cellular signaling network, creating a favorable environment for the replication and persistence of the virus. For example, HSV-1 proteins, such as ICP0, ICP4, and ICP27, can affect host gene expression and block cellular signaling pathways, including p53, NF-κB, and MAPK-mediated signaling pathways. These alterations in cell signals can lead to dysregulation of cell proliferation, inhibition of apoptosis, and evasion from immune surveillance, contributing to the survival and growth of viruses and potentially transformed cells (33).

On the contrary, our study found no significant causal association between HSV-2 infection and HNC, including oral and oropharyngeal cancer. This finding was in agreement with several previous investigations (34). Thompson et al. conducted a systematic review and meta-analysis of the existing literature and believed that there was insufficient evidence to support the direct link between HSV-2 infection and HNC. Furthermore, Chen et al. failed to unravel a significant association between HSV-2 seropositivity and the risk of oropharyngeal cancer in a large prospective cohort study. Overall, combined with these studies, our study demonstrated that different from HSV-1, HSV-2 infection might not be an important dangerous factor for HNC (35).

It was worth noting that HSV-1 and HSV-2 exhibited different associations with HNC, which might be attributed to their different biological characteristics and modes of transmission. HSV-1 mainly infects oral and oropharyngeal mucosa, while HSV-2 mainly affects genital and anal regions. Different anatomic regions of infection may lead to different carcinogenic potentials of these two viruses. Additionally, differences in viral gene expression, immune response, and cytotaxis may also contribute to different associations (36).

However, several studies have reported conflicting results on the relationship between HSV-1 and HNC. For instance, a case-control study by Roberts et al. reported no significant association between HSV-1 seropositivity and the risk of oropharyngeal cancer. Likewise, Brown et al. failed to observe a significant relationship between HSV-1 infection and the risk of HNC in a population-based cohort study. These contradictory results might be attributed to diverse factors, including study design, sample size, population characteristics, and different HSV-1 detection methods (37).

Taken together, our research result was basically consistent with prior studies, that was, HSV-1 infection was a risk factor for HNC, especially oral and oropharyngeal cancer. Our study did not show a causal association between HSV-2 infection and HNC, which was supported by the existing literature, highlighting the importance of distinguishing these two HSV types in evaluating their potential roles in carcinogenesis.

According to the literature review, this is the first Mendelian randomization study on HSV infection and the risk of head and neck cancer. This article analyzes the association between the two at the genetic level. However, there are still some limitations in this study. First, the GWAS data on HSV are limited, and it is impossible to use multiple data sets to verify our results. Second, the study population is all Europeans, so it is impossible to predict the relationship between HSV and head and neck cancer in other populations.

## 5 Conclusions

In this study, MR analysis was adopted to assess whether HSV infection was causally linked to the development of HNC. We noted a significant causal relation between HSV-1 infection and the progression of HNC, particularly oral and oropharyngeal cancer, but no such causal relation was found between HSV-2 infection and HNC.

## Data availability statement

The datasets presented in this study can be found in online repositories. The names of the repository/repositories and accession number(s) can be found in the article/**Supplementary Material**.

## Ethics statement

The manuscript presents research on animals that do not require ethical approval for their study.

## Author contributions

MY: Data curation, Methodology, Software, Visualization, Writing – original draft. LX: Methodology, Project administration, Writing – review & editing. MG: Formal analysis, Investigation, Data curation, Writing – review & editing. RF: Data curation, Formal analysis, Writing – review & editing. RS: Data curation, Formal analysis, Methodology, Writing – review & editing. H-CF: Conceptualization, Formal analysis, Methodology, Project administration, Writing – review & editing. L-LF: Formal analysis, Funding acquisition, Investigation, Validation, Writing – original draft. SB: Data curation, Formal analysis, Validation, Writing – review & editing.
